# Peroxiporins in Triple-Negative Breast Cancer: Biomarker Potential and Therapeutic Perspectives

**DOI:** 10.3390/ijms25126658

**Published:** 2024-06-17

**Authors:** Anita Bijelić, Tajana Silovski, Monika Mlinarić, Ana Čipak Gašparović

**Affiliations:** 1 Department of Biology, Josip Juraj Strossmayer University of Osijek, 31000 Osijek, Croatia; anitabijelic0511@gmail.com; 2 Department of Oncology, University Hospital Centre Zagreb, 10000 Zagreb, Croatia; tsilovsk@kbc-zagreb.hr; 3 School of Medicine, University of Zagreb, 10000 Zagreb, Croatia; 4 Division of Molecular Medicine, Ruđer Bošković Institute, 10000 Zagreb, Croatia; monika.mlinaric@irb.hr

**Keywords:** triple-negative breast cancer, aquaporins, peroxiporins

## Abstract

Triple-negative breast cancer (TNBC) remains one of the most challenging subtypes since it is initially characterized by the absence of specific biomarkers and corresponding targeted therapies. Advances in methodology, translational informatics, genomics, and proteomics have significantly contributed to the identification of therapeutic targets. The development of innovative treatments, such as antibody–drug conjugates and immune checkpoint inhibitors, alongside chemotherapy, has now become the standard of care. However, the quest for biomarkers defining therapy outcomes is still ongoing. Peroxiporins, which comprise a subgroup of aquaporins, which are membrane pores facilitating the transport of water, glycerol, and hydrogen peroxide, have emerged as potential biomarkers for therapy response. Research on peroxiporins reveals their involvement beyond traditional channeling activities, which is also reflected in their cellular localization and roles in cellular signaling pathways. This research on peroxiporins provides fresh insights into the mechanisms of therapy resistance in tumors, offering potential avenues for predicting treatment outcomes and tailoring successful TNBC therapies.

## 1. Introduction

Since their discovery, our knowledge of aquaporins (AQPs) reveals the intricate regulation and diverse functions governing their interactions. Agre described them as the cell’s plumbing system [1], yet their role and function in the cell are far from being as straightforward. Today, we understand the mechanism governing water transport, which is highly specific for water molecules due to the selectivity filter within the pore [2]. In addition, AQPs not only facilitate water transport, but also glycerol, ions, urea, lactic acid, and hydrogen peroxide [3]. Interestingly, glycerol can hinder the flow of water through aquaporins. Unlike water, glycerol flows through aquaglyceroporins by rotational movements, highlighting the intricate nature of these pores and their role in molecular transport within biological systems [4]. Moreover, AQP6 deviates from the typical function of aquaporin as a water channel, but functions as an anion channel. This change in function arises from the critical substitution of glycine (Gly-57) to asparagine residue (Asn-60), the position corresponding to Gly-57 [5]. In addition to water, glycerol, and ions, and due to high structural similarities to water regarding size, dielectric properties, and capacity to form hydrogen bonds, H_2_O_2_ is also a substrate for AQPs [6]. These AQPs involved in channeling H_2_O_2_ are named peroxiporins [7]. The ability of aquaporins to transport several substrates widens their function to include not only the regulation of cellular and tissue water homeostasis but also cell proliferation, migration, and adhesion [8]. Following these functions, it is not surprising that AQPs have been implicated in various non-communicable diseases, including cancer [8]. Furthermore, the regulation of AQPs and their response to various stimuli contribute an additional layer of complexity to understanding the pathways influenced by AQPs. An example of this complexity lies in the most prominent regulatory mechanism of AQP function, which involves their subcellular translocation from intracellular vesicles to the membrane in response to hormonal stimuli [9]. Hence, investigating the role of AQPs in tumorigenesis presents a challenge, as their role and mechanisms of their regulation are far from being straightforward. Furthermore, new functions and interactions are emerging, complicating our understanding of their role in cancer development and therapy resistance.

The worldwide cancer mortality rate is 8.2 million people per year and it is expected that 13.1 million people will die of cancer by 2030, thereby overtaking cardiovascular disease as the leading cause of death in humans [10,11]. Breast cancer is the leading cause of cancer death in women, with 2.3 million cases annually [10,12].

Because of its molecular and clinical heterogeneity, the personalization of breast cancer diagnosis and treatment is necessary. In the era of molecular testing, conventional prognostic factors, such as lymph node metastasis, tumor size, and histologic tumor grade, are no longer sufficient to personalize treatment and diagnosis. Molecular prognostic and predictive biomarkers increase the knowledge of tumor characteristics, enabling oncologists to predict tumor aggressiveness and invasiveness. The critical role of molecular biomarkers is to avoid the undertreatment, mistreatment, and overtreatment of those who will not benefit from therapies [12]. Consequently, new biomarkers are needed to better characterize the disease and to apply effective therapy. 

Currently, markers in breast cancer diagnostics are determined routinely by immunohistochemistry (IHC) and include hormone receptors (estrogen receptor (ER) and progesterone receptor (PR)), human epidermal growth factor receptor 2, HER2, and Ki67. Based on the marker profile, breast cancer can be classified into four surrogate subtypes (Table 1). Breast cancer with a low or negative ER and PR without expression or amplification of the HER2 receptor is often defined as triple-negative breast cancer (TNBC).

Tumor protein p53 (TP53), *Breast Cancer Gene 1* (*BRCA1*), and *Breast Cancer Gene 2* (*BRCA2*) play important roles in breast cancer development [12,13]. BRCA1 and BRCA2 are tumor suppressor genes, where mutations cause inefficient DNA repair, further increasing the mutation rate and supporting tumor development [12]. *BRCA1* and *BRCA2 (BRCA1/2)* gene mutations are the most encountered cancer predisposition genes and are present in less than 10% of breast cancer cases and around 10–20% of TNBC patients [14]. *BRCA1/2* have an essential role in cycle checkpoints and DNA repair via the homologous recombination pathway [15].

There is an ~80% overlap between the triple-negative and intrinsic basal subtypes, but the triple-negative subtype also includes some special histological types [12,16]. Due to the overlapping features, basal-like breast cancer can be misinterpreted as TNBC (77% of them really are TNBC) [17]. Vice versa, 71–91% of TNBCs are basal-like, and the final observation is that both types of breast cancer overlap and are classified differently. Additionally, each of these cancers has unique clinical outcomes, phenotypes, and pharmacologic sensitivities [17].

## 2. Triple-Negative Breast Cancer

TNBC accounts for approximately 15–20% of all breast cancers. It is most common in premenopausal women younger than 40 years of age and individuals with inherited gene mutations that primarily affect *BRCA1* and/or *BRCA2* genes. TNBC is characterized by aggressive clinical behavior and a poor prognosis, with a shorter survival of these patients compared with other breast cancers [14,18]. The mortality rate is 40% in the first five years after diagnosis, and it is mostly due to high invasiveness and distant metastases (the median survival time for metastatic TNBC is only 13.3 months) [14,18]. One of the issues in treating TNBC is the lack of therapeutic targets [19], making local treatments, such as surgery and radiotherapy, as well as chemotherapy-based systemic therapy, the mainstays of TNBC treatment [20]. 

**Figure 1:**
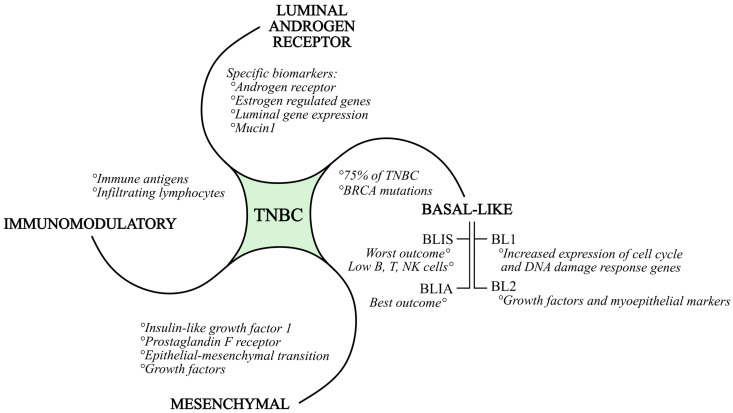
Subtypes of triple-negative breast cancer, together with their characteristics.

The intense genomic and transcriptional heterogeneity of TNBC is responsible for the complexity of defining appropriate molecular targets in preclinical studies. Burstein et al. proposed, after RNA and DNA profiling, a four-type classification of TNBC (Figure 1): basal-like immuno-suppressed (BLIS), basal-like immuno-activated (BLIA), mesenchymal (MES), and luminal androgen receptor type (LAR) [21]. Lehmann et al. redefined the TNBC subtypes as follows: basal-like (BL1 and BL2), immunomodulatory (IM), MES, and LAR [22]. For each subtype, the following is to be mentioned: BLIS tumors have the worst outcome, while BLIA tumors have the best outcome; the LAR subgroup is defined by specific biomarkers and targets, such as the androgen receptor MUC1 and several estrogens regulated genes. The MES subgroup is defined by IGF1, which is the prostaglandin F receptor [21]. A characteristic of the BL1 subtype is the increased expression of cell cycle and DNA damage response genes. The BL2 subtype is defined by growth factor signaling and myoepithelial markers. As already mentioned, the basal-like subtypes constitute 75% of TNBC. According to studies, basal-like immunosuppressed TNBC subtypes have lower numbers of B cells, T cells, and natural killer cells, resulting in a poorer prognosis.

In general, all *BRCA1* and *BRCA2* mutations are associated with basal-like gene patterns [10]. *BRCA*-mutated (*mBRCA*) breast cancer is more probable in individuals with a familial background of breast cancer, those who are younger, and those experiencing concurrent or successive occurrences of breast and ovarian cancer on the opposite side [23]. The IM subtype is composed of genes that encode immune antigens, cytokine, and nuclear immune signal transduction pathways, and likely represents gene expression from both tumor cells and infiltrating lymphocytes. The MES subtype exhibits increased expression of the epithelial–mesenchymal transition and growth factor genes. Luminal gene expression is part of the LAR subtype, and the androgen receptor is a driver of the LAR subtype. Cell lines of each subtype show different sensitivities to the alkylating agent cisplatin. All mentioned subtypes may be targeted with efficient TNBC therapy in the future [21,22].

There are interesting data about the connection between TNBC and the tumor microenvironment (TME), which contains the extracellular matrix (ECM), vascular endothelial growth factors, tumor-associated macrophages (TAMs), tumor-infiltrating lymphocytes (TILs), and other molecules important for tumor growth and migration [10]. Genetic modifiers, developmental pathways, growth factors, chemokines, exosomes, epigenetic regulators, and microRNAs control the TME. Expertise on the TME and its regulators will be beneficial in shaping targeted therapies for TNBC [24].

Currently, following the initial work-up, which involves radiological assessment of the primary tumor using mammography, MRI, and/or ultrasound, the diagnosis of TNBC is confirmed through the histopathological evaluation of tumor specimens obtained via a core needle biopsy. Disease staging is then conducted by assessing the regional lymph node involvement and determining the presence of distant metastases [16].

A blood-based liquid biopsy is one of the diagnostic methods that may be considered for TNBC diagnosis in the future. A blood-based liquid biopsy analyzes the presence of circulating tumor cells (CTCs), tumor-derived extracellular vesicles (exosomes), and circulating tumor nucleic acids (ctNAs), which include circulating tumor DNA (ctDNA) and microRNAs (miRNAs). The amount of ctDNA in the bloodstream depends on the size of the tumor or metastases burden, which leads to the assumption that a higher ctDNA concentration increases the probability of tumor metastasis existence [25].

## 3. TNBC Therapy

TNBC has traditionally been considered a heterogenous disease with poorly understood behavior, thereby delaying the development of targeted therapy compared with other breast cancer subtypes. Due to the lack of therapeutic targets, such as ER and PR expression and HER2 overexpression, as well as the absence of actionable biomarkers and molecular tumor growth drivers, the treatment of TNBC has been and continues to be mainly based on chemotherapy as the standard of care. Significant progress in TNBC treatment has been achieved by optimizing chemotherapy delivery by considering the selection, dosing, and sequencing of cytotoxic drugs, as well as applied therapy protocols [26].

In early, localized disease, the improvement in outcome has been based on the escalation of systemic therapy, and significant effort has been undertaken to switch from the historically adapted approach of postoperative adjuvant systemic therapy toward therapy delivered preoperatively in the neoadjuvant setting. Systemic therapy in this setting is composed of anthracycline- and taxane-based chemotherapy combinations [16,27], decreasing the mortality rate by ~38% in patients younger than 50 years and ~20% in patients aged 50–69 years old [28]. The efficacy of therapy varies greatly between subtypes. TNBC patients with basal-like subtypes respond better to chemotherapy in comparison with the mesenchymal and luminal androgen receptor subtypes [29,30]. The addition of a platinum compound to the standard neoadjuvant therapy backbone has improved the pathological complete response (pCR) rates, which can be attributed to the known TNBC sensitivity to DNA-damaging drugs [31,32,33,34,35]. The described benefit was translated into the improvement of long-term outcomes, like event-free survival (EFS), as well as disease-free survival (DFS) [36].

The neoadjuvant strategy has enabled in vivo assessment of tumor sensitivity to the systemic treatment, and therefore, accelerated novel drug development, together with the de-escalation of surgery [37,38]. It has also allowed for making decisions on postoperative adjuvant therapy escalation or de-escalation, depending on the individual response to the neoadjuvant therapy [39,40]. Patients with an incomplete response to neoadjuvant therapy experience higher recurrence and mortality rates, which are amplified with the extent of residual disease measured by the pathologist using the Residual Cancer Burden (RCB) index [41,42,43,44,45]. Patients who do not achieve a pCR should be offered adjuvant therapy after surgery as a therapy escalation modality, while patients who achieve a complete response are candidates for de-escalation strategy and could be spared the toxicity of additional postoperative therapy. The new potential biomarkers for identifying high-risk patients among those who do not achieve a pCR include the detection of minimal residual disease by circulating free DNA and the assessment of tumor-infiltrating lymphocytes in residual disease [45,46,47]. 

Patients with early TNBC treated with neoadjuvant chemotherapy alone who do not achieve a pCR should be offered adjuvant capecitabin due to overall survival (OS) improvement confirmed by the Asian Create-X study [35]. To improve the outcome of early TNBC, the search for new therapy approaches is intensively ongoing, with new targets emerging. Immunotherapy provides a good approach to targeting specific molecules in immune and cancer cells. One such target is the programmed cell death protein ligand 1 (PD-L1) [18,48], which is expressed on tumor cells and binds to the programmed cell death protein 1 (PD-1) expressed on the T cell surface [49]. The binding of PD-L1 to PD-1 enables tumor cells to modulate the activity of immune cells (T cells in particular) in the tumor [50], and therefore, it is not surprising that PD-L1 and/or PD-1 are highly expressed in TNBC and are associated with histological-grade and tumor-infiltrating lymphocytes [18,48]. Several clinical trials demonstrated significant improvement in the pCR with a combination of chemo- and immunotherapy [51,52]. The anti-PD-1 monoclonal antibody and checkpoint inhibitor pembrolizumab is currently the standard in combination therapy with anthracycline, taxane, and carboplatin chemotherapy for high-risk (stage II and III) early TNBC according to the Keynote-522 trial, regardless of PD-L1 expression [52,53]. In addition to pembrolizumab, other checkpoint inhibitors, such as atezolizumab [54], cemiplimab [55], and durvalumab, have been explored in the treatment of early TNBC. These trials have demonstrated the benefits of immunotherapy, contributing to increased pCR rates and prolonged invasive DSF (iDSF) [56].

Another strategy arises from tumor development, during which the accumulation of mutations occurs due to DNA damage, evading multiple repairing mechanisms [57]. Poly-ADP-ribose-polymerase (PARP) is a crucial protein involved in DNA repair mechanisms, such as homologous recombination and non-homologous end-joining deficiency-based repair, as well as base excision repair [58]. PARP inhibition is associated with the induction of DNA damage and subsequent destruction of *BRCA*-mutated cancer cells [58]. Therefore, PARP inhibitors, such as olaparib, are a therapy option in BRCA-mutated patients who are upfront surgically treated for high-risk TNBC or those with residual disease after neoadjuvant treatment of *BRCA*-mutated patients [23]. 

**Figure 2:**
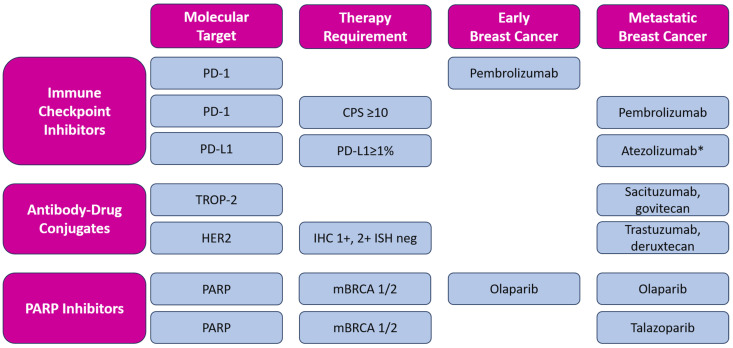
Overview of targeted therapy for early and metastatic triple-negative breast cancer with included molecular expression for each molecule. Magenta boxes: header row and column, blue boxes: molecular targets, categorized by types of therapy and including the specific requirements for the use of each therapy in early and metastatic breast cancer; All listed medicines are approved by EMA and FDA, except * atezolizumab, which is approved by EMA only [61,62]. CPS—Combined Positive Score; IHC—immunohistochemical score; ISH—in situ hybridization.

In addition to novel approved therapeutic options, such as immune checkpoint inhibition and targeted therapy (including PARP inhibition), antibody-drug conjugates (ADCs), such as sacituzumab-govitecan (SG) and trastuzumab-deruxtecan (T-DXd), have emerged as a new therapeutical approach, contributing to a modest improvement in the prognosis of metastatic TNBC. ADCs are new antineoplastic agents consisting of a monoclonal antibody conjugated to a cytotoxic payload by a linker. In addition to these two ADCs, other new molecules are being investigated in metastatic TNBC, such as datopotamab-deruxtecan [59] and patritumab-deruxtecan [60]. The described targeted therapies approved by FDA and EMA are listed in Figure 2.

Furthermore, along with the aforementioned strategies, studies on AKT inhibitors (e.g., capivasertib, ipatasertib), in combination with chemotherapy, are ongoing. Other promising therapies include androgen receptor antagonists, JAK1/2 inhibitors, and PI3K inhibitors for various subtypes, all of which are currently undergoing clinical trials [63,64].

The development of genomic, transcriptomic, and proteomic profiling of both cancer cells, as well as the cancer microenvironment, has resulted in groundbreaking classifications of TNBC, revealing distinct oncogenesis drivers [21,22,65,66]. As usual, novel therapies initially demonstrated efficacy in the metastatic disease setting before swiftly advancing to the neoadjuvant or adjuvant treatment of early breast cancer [39,53].

Unfortunately, in the setting of metastatic TNBC, therapy resistance will eventually emerge. Multiple resistance mechanisms have been identified across different therapeutic options. The continuous pursuit of new therapeutic strategies for pretreated patients aims to overcome such resistance challenges [67].

## 4. Aquaporins

The aquaporin protein family consists of 13 members (AQP0–AQP12), with isoforms expressed in specific tissues through unique combinations [68]. They are pores that facilitate the transport of water and other small molecules across the membrane [8]. Affinity toward different substrates categorizes them into distinct groups: orthodox aquaporins (AQP0, AQP1, AQP2, AQP4, AQP5, AQP6, and AQP8) primarily facilitate water transport and aquaglyceroporins (AQP3, AQP7, AQP9, and AQP10) channel glycerol, while S-aquaporins are grouped due to their exclusive intracellular localization (AQP11 and AQP12) [8]. In addition to their primary substrates, specific aquaporins, including AQP1, AQP3, AQP5, AQP8, AQP9, and AQP11, also function as channels for hydrogen peroxide. The importance of channeling hydrogen peroxide is recognized by naming them peroxiporins [69]. 

**Figure 3:**
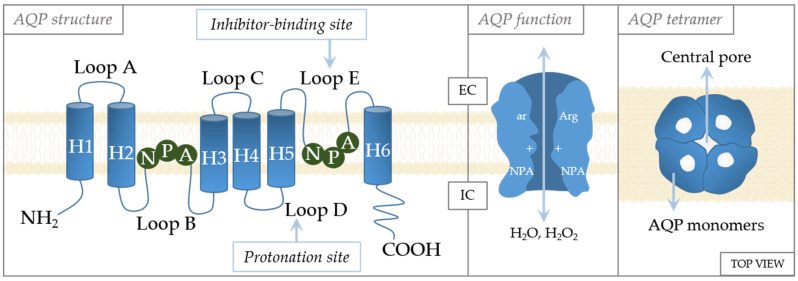
Schematic structure of the aquaporin monomer. The monomer consists of six transmembrane α-helices (H1–H6) connected with five loops (loops A–E). The cellular orientation of the N- and C-termini are indicated as being on the extracellular (EC) or intracellular (IC) side, as well as regulation sites (inhibitor-binding site and protonation site). Regulatory amino acids: A—alanine, N—asparagine, P—proline, Arg—arginine, ar—aromatic amino acid.

The crystal structures of AQPs reveal high similarities in their structures (Figure 3). They are tetramers, with each monomer functioning independently of the others and forming the central pore [70]. Each monomer consists of six transmembrane α-helices with five loops (loops A–E) in between [71]. Loops B and E are of special importance to aquaporin gating [72]. These loops have an NPA motif (asparagine–proline–alanine) that are located in the center of the channel [73]. The specificity of channeling is dictated by these motifs that form an hourglass structure [74], thereby providing maximized permeability and optimized hydrodynamic transport [75]. Another constriction site, the aromatic/arginine (ar/R) constriction site, is near the extracellular side of the pore [76]. Residues at the ar/R site are variable in the AQP family, being the factor that causes AQPs to differ in size and hydrophobicity, as well as channel selectivity [77,78]. The importance of this site in selectivity is supported by the study of Beitz et al., which showed that point mutation in the ar/R region dictates the selectivity of the channel [77]. In addition, the ar/R region is wider in aquaglyceroporins than in aquaporins [76]. 

Although very similar in structure, they exhibit distinct characteristics and variations in the ar/R constriction site, which affects the pore size and dictates their permeability. AQP1 is a 28 kDa protein when unglycosylated, or approximately 40 kDa when glycosylated [79]. Early research on erythrocytes revealed that AQP1 is a homotetramer with one glycosylated subunit [80,81]. At its C-terminus, approximately 4.3 kDa in size, AQP1 contains motifs for the Ca^2+^ binding site, which regulates gating via binding cyclic guanosine monophosphate (cGMP) [80]. The ar/R constriction site of AQP1 comprises phenylalanine and histidine, which are common residues for orthodox AQPs, and cysteine, which is specific to AQP1 [82]. Aquaglyceroporin AQP3 has its gating regulated by pH [83], with pH-sensitive residues (His53, His154, Tyr124, and Ser152) located in its extracellular loops [84]. AQP3 is also sensitive to divalent cations, such as Ni^2+^ or Co^2+^ [85]. The residues Trp128, Ser152, and His241 located on its extracellular side were identified as targets for the inhibition of these two divalent cations [84,86]. While AQP3 is known to be permeable to glycerol, there are conflicting data on its permeability to urea, which could be attributed to variations in assays used to measure this permeability [82]. AQP5 is a 265-amino-acid-residue-long water-permeant aquaporin [71]. Interestingly, a high-resolution X-ray structure showed the presence of phosphatidylserine in the central pore [87]. However, the role of phosphatidylserine in this context remains unclear. Most of the studies focused on trafficking as a mode of AQP5 regulation and identified phosphorylation sites that are thought to have a role in trafficking. Two of these phosphorylation sites, Ser152 and Ser156 [88,89], are located in loop D, while Thr259 is located in the C-terminus [90]. The C-terminus also harbors Ser156, which is a target for protein kinase A phosphorylation, which triggers the Ras signaling pathway [91].

The central pore is formed due to the tetrameric assembly of AQP monomers, leaving a pore in the middle of the structure. The first reports implied that due to its hydrophobic nature, the central pore conducts gases [92]. At first, there were implications that ions are also transported through central pore, [92,93], but now we know that CO_2_, NO, Na^+^, K^+^, and Cs^+^ permeate through the central pore of AQP1 [94,95]. The permeation of cations is regulated by cGMP, which causes conformational changes of loop D [94]. Furthermore, molecular dynamics showed that CO_2_ can permeate easier through the central pore than through a monomer of AQP5, and it can also permeate through AQP1 and AQP4 [96,97].

Due to their involvement in the movement of these diverse molecules across the membrane, aquaporins play a crucial role in cellular and tissue water homeostasis, migration, cell–cell adhesion, and proliferation [71,98,99,100]. Furthermore, aquaporins play a role in various pathological conditions that stem from the dysregulation of their expression and/or pore activity. This dysregulation has cascading effects on the movement of water, glycerol, and hydrogen peroxide, contributing to the complexities of these pathological scenarios.

In breast mammary glands, two peroxiporins are found, specifically AQP1 and AQP3. AQP3 is highly expressed on the basolateral membranes of mammary ducts and glands [101], while AQP1 has notably lower expression [102]. In breast cancer, the expression of both AQP1 and AQP3, along with newly occurring AQP5, is elevated [102,103]. Here, it should be noted that AQP5 is absent in normal, non-tumorigenic mammary tissue, but appears in cancer, reaching its highest levels in metastatic breast cancer [103]. These changes in AQP expression patterns highlight the importance of AQP-tissue-specific expression patterns as potential biomarkers of the malignant transformation of breast tissue. Furthermore, increased AQP1 expression in breast cancer is emerging as a potential prognostic marker, particularly for the basal-like phenotype of breast carcinoma, and correlates with a poorer prognosis for patients [104]. The association of AQP1 with hypoxia-induced angiogenesis, independent of VEGF [105], suggests that its increased level in tumors provides a strategic advantage in nutritional supply and supports growth. Moreover, the effects of AQP1 overexpression extend beyond this, as the immunostaining of tumor tissue reveals that AQP1 positivity moves from membrane-only to the cytoplasmic positivity, an implication which still remains to be elucidated [104,106]. Furthermore, AQP1 positivity showed stratification in tumor tissue, with stronger expression observed at the invasive front of the tumor, indicating an important role in tumor growth [104].

## 5. Peroxiporins as Biomarkers for TNBC

The initial studies on aquaporins primarily focused on their expressions in tumor tissue and on their roles in cellular functions within cellular models. In breast cancer, AQP1 [107], AQP3 [108], and AQP5 [109] were found to be upregulated. The altered cellular functions that result from their upregulation were comprehensively reviewed in [98,103,110,111]. Notably, these three aquaporins are also regulated by trafficking, where they are translocated to the membrane in response to hormonal stimuli through G-protein-coupled receptors [112]. AQP5 localization within the tumor tissue correlates with a study on MDCK cells, indicating no significant differences in overall AQP5 levels, but rather an AQP5-intensified distribution at the site of migration [113]. AQP5 has also been associated with the downregulation of junctional proteins, independent of Ras activation (Ser156 residue) triggering cell detachment, which is a crucial event in cancer dissemination [113]. Similarly, a decrease in junction proteins was also observed for AQP1, while AQP3 affected different junctional proteins, indicating the importance of studying these AQPs together and considering their relative ratios [98,113]. The intricate interplay between numerous proteins contributing to cancer progression is reflected in the regulation of AQP1 by PKC [112,114], which is often overexpressed in cancer, and thus, a target for cancer therapy [115]. The inhibition of AQP1 by miR-3194-3p reduced the migration, proliferation, and apoptosis of MDA MB 231 and MCF7 cells [116]. These examples illustrate just a few facets of AQP function in (cancer) cells. For these reasons, aquaporins are emerging as novel biomarkers in the management of breast cancer, as well as other types of cancers. Their potential applications in diagnostics are summarized in Table 2.

Early research reported that AQP1 expression is associated with a particularly aggressive subgroup of basal-like breast carcinomas and localizes predominantly in the membrane, but also in the cytoplasm [104]. The AQP1-positive carcinomas also exhibited increased membrane and cytoplasmic AQP1 positivity in all adjacent myoepithelial cells, further supporting AQP1 as a strong marker of the poor outcome [104]. Furthermore, localization of AQP1 was also shown to be a determining factor in prognosis. In benign lesions and in situ ductal carcinomas, AQP1 was localized primarily in plasma membranes, whereas in invasive ductal carcinoma cells, it was found in the cytoplasm and correlated with breast cancer invasiveness [119]. Moreover, AQP1 expression is elevated in blood vessels of breast cancer tissues. This is supported by the in vitro part of the same study, which showed that AQP1 is induced by estrogen, highlighting the role of estrogen in angiogenesis [137].

A study of AQP3 and AQP5 expression patterns in TNBC patients indicates that the overexpression of these two peroxiporins is strongly associated with tumor aggressiveness and poor prognosis. Both AQP3 and AQP5 were found in the cytoplasm and membrane of tumor cells [126,127], similar to APQ1. Furthermore, an analysis of the expression profile of aquaglyceroporins, which was correlated with immunohistochemical staining, revealed that in addition to AQP3, AQP7 and AQP9 are also expressed in normal breast epithelia but are increased in invasive ductal carcinoma, with AQP7 and AQP9 localized only intracellularly [127]. This study concluded that a unique combination of AQP expression could serve as a biomarker for personalized anticancer therapy [127]. Interestingly, AQP7 regulates lipid, GSH, and urea/arginine metabolism, thereby regulating the response to stress of cancer cells [138].

AQP5, along with AQP1 and AQP3, is strongly associated with breast cancer malignancy, correlating with the tumor grade. It is more frequent in hormone-receptor-negative tumors, and strongly correlated with HER2-positive tumors [139]. This increase in AQP5 expression is in some part due to gene amplification [139]. Several studies confirmed through immunolabeling that AQP5 increases with the malignancy of the disease and correlates with a poor prognosis [103,140,141]. Examining the localization of AQP5 within the tumor tissue, it is found at the invasive front of the tumor and decreases in necrotic parts [126]. 

These studies suggest the possible use of AQPs as biomarkers for predicting the prognosis. We are now aware that AQPs should be studied together rather than individually, highlighting the interplay between isoforms of these channels in the regulation of cellular processes that lead to pathology. 

## 6. Peroxiporins as Potential Targets in TNBC Therapy

As our understanding of the pathways influenced by aquaporin activity increases, the focus is now shifting toward the effects of aquaporins on cancer therapy. Moreover, accumulating evidence suggests that aquaporins modulate cancer therapy resistance, with the effect being specific to each involved aquaporin. The story gains complexity when considering the tumor microenvironment, where interactions with adipocytes contribute to the intricate story. Furthermore, specific aquaporins can have different roles in the cancers of different origins [99]. In this context, we focused on peroxiporins, which have demonstrated a significant role in breast cancer, with highlights summarized in Table 3.

Among AQPs, AQP1 emerged as a promising target to elucidate mechanisms of tumor progression and therapy resistance. As discussed in the previous section, AQP1 showed potential as a biomarker of breast cancer aggressiveness [119], linking its expression to increased sensitivity to the anthracycline therapy of invasive ductal carcinoma patients [117]. These findings combined suggest that the subcellular localization of AQP1 [119] could reflect its function in anthracycline sensitivity. In MDA-MB-231 cells, AQP1 competes with GSK3β for binding to β-catenin, stabilizing the latter by inhibiting ubiquitination. This interaction occurs via the C-terminus of AQP1 and results in increased sensitivity to epirubicin by enhancing topoisomerase IIα activity [117]. Additionally, the role of cytoplasmic AQP1 is further supported by a study on the MDA-MB-231 cell line. The overexpression of AQP1 in these cells showed that AQP1 coprecipitated with Annexin A2, localizing to the perinuclear Golgi region and inducing Golgi apparatus extension [118]. Furthermore, cytosolic AQP1 forms a complex with cytosolic free Rab1b protein and Annexin A2, triggering the secretion of ICAM1 and CTSS, and thus, inducing migration and invasion [118]. These findings suggest that AQP1 serves not only as a biomarker for anthracycline sensitivity but may be a potential target for preventing or reversing this resistance.

Another peroxiporin, AQP3, is a candidate for use in the management of breast cancer chemotherapy resistance. Treatment with 5’fluorouracile, 5’-DFUR, and gemcitabine, but not cisplatin, caused cell cycle arrest in MCF7, which is a response that was reversed by silencing AQP3 [124]. Yet, in the triple-negative cell line MDA-MB-468, only gemcitabine caused an increase in AQP3 mRNA and in cell volume [124]. In contrast, in another study, AQP3 silencing in MDA-MB-231 cells reduced the proliferation and migration, as well as increased the sensitivity to 5-fluorouracil, while leaving adhesion unaffected [125]. Although there is still not enough evidence for the role of AQP3 in chemotherapy resistance in TNBC, accumulating evidence suggests that AQP3 can have a role in the resistance to nucleotide analogs in estrogen-positive tumors, which is not surprising, as estrogen-binding sites are found in the promoter regions of the AQP3 gene [108]. As is the case with AQP1, there is a question whether AQP3 localization in the cancer cell cytoplasm could be of importance for its role in cancer aggressiveness.

Along with the aforementioned AQP1 and AQP3, AQP5 is strongly associated with breast cancer malignancy [126]. Studies on the hormone-positive cell line MCF7 with doxorubicin-sensitive and -resistant variants, suggest that at least several peroxiporins are implicated in breast cancer resistance. Especially interesting is the upregulation of AQP5 expression in its doxorubicin-resistant variant. The silencing of AQP5 in this resistant strain resulted in decreased proliferation and induction of apoptosis [131]. Yet, in the AQP5-overexpressing MDA-MB-231 spheroids doxorubicin, a combination of doxorubicin, cisplatin, and 5-FU or a combination with the Ras inhibitor salirasib, but not cisplatin and 5-FU alone, reduced the spheroid size and viability, indicating the role of AQP5 in the sensitivity to doxorubicin [132]. Furthermore, AQP5 with a mutation for the Ras binding site S156A did not affect the spheroid size, suggesting that Ras could have a role in the increased sensitivity to doxorubicin [132]. AQP5 also positively regulates Rac1, which is a protein that regulates lamellipodia; modulates cell growth by the activation of NFkB, JNK, and p38 mitogen-activated protein kinase (MAPK); and also contributes to ROS production [133]. In colorectal cancer cells, the knockdown of AQP5-sensitized cells to 5-FU acts through the inhibition of Wnt-β catenin signaling [142].

Studies on the role of aquaporins/peroxiporins in breast cancer and therapy resistance usually focus on an individual aquaporin, overlooking potential changes and/or interactions with other aquaporins. However, presenting changes in aquaporins relevant to breast cancer in parallel could highlight the necessity of studying these aquaporins together. Investigating AQP1, AQP3, and AQP5 together may yield deeper insights into their interactions and collective contributions to therapy resistance in breast cancer.

Finally, the evidence on breast cancer tissue and on breast cancer cell lines implies peroxiporin involvement in the development of cancer and therapy resistance. Their intricate role in cell adhesion and change in cell localization in cancer highlights the need to study them in more detail and to obtain a wider picture by introducing more than one peroxiporin in the study due to their overlapping functions and differential roles in different types of cancer.

## 7. Future Perspectives

Despite their straightforward role at first sight, aquaporins are complex and multifaced proteins. Emerging research suggests that the interplay between various aquaporins can significantly influence cellular responses, which is crucial for cancer treatment. In cancer, there is a disbalance of aquaporins, not only in aquaporins present in the non-tumorigenic tissue but also newly expressed aquaporins. Current evidence positions aquaporins as biomarkers for the cancer progression and as important players in defining therapeutic responses. Consequently, future research should focus on all isoforms actively expressed in cancer cells. This could open the door for personalized therapies based on the aquaporin expression profile and possible additional therapeutic strategies to target aquaporins, thereby sensitizing cancer cells to treatment.

## 8. Conclusions

While aquaporins were at first described as the “plumbing system of the cell”, their role in cellular processes and signaling pathways is very complex and multidimensional. In addition, our understanding of the biology and specific molecular characteristics of TNBC is improving, where we now recognize subtypes of this malignant disease. These evolving fields present new opportunities for improving TNBC therapy. AQP1, AQP3, and AQP5 have emerged as promising biomarkers for breast cancer malignancy, with studies implying their role in the effectiveness of cancer chemotherapy. These AQPs have the potential to support TNBC chemotherapy effectiveness and, as such, have a great potential as biomarkers for therapy selection in TNBC. Nevertheless, the previous studies imply the necessity to study peroxiporin biology, integrating the new therapeutic approaches in TNBC to elucidate their role and eventually incorporate them into clinical practice.

## Figures and Tables

**Table 1:** Classification of breast cancer based on immunohistochemical markers.

Breast Cancer Type		ER	PR	Ki67	HER2
Luminal A		+	+	Low	−
Luminal B	HER2−	+	+/−	High	−
	HER2+				+
HER2-positive		−	−	−	+
Triple-negative		− or low	−	−	−

**Table 2:** Aquaporins and breast cancer diagnostics.

Peroxiporin	Expression in BC	Cytoplasmic Localization in BC	*Cancer Type* *(Except BC) Increased*	Reference
AQP1	Increased in aggressive basal-like BC	+	Brain, colon, lung, ovarian, colon cancer, pancreatic, prostate cancer	[117,118,119,120,121,122,123]
AQP3	Increased with BC malignancy	+	Colon, lung, prostate, esophageal and oral squamous cell carcinoma, hepatocellular, pancreatic adenocarcinoma	[108,120,124,125,126,127,128]
AQP4	−	N/A	Brain, gastric, lung, pancreatic, thyroid gland cancer	[120,123,129,130]
AQP5	Not present in normal breast epithelia; associated with BC malignancy	No data, but localized on the invasive front of the tumor	Ovary, prostate, gastric, pancreatic cancer	[123,126,131,132,133,134,135]
AQP6	−	N/A	Gastric, ovarian cancer	[121,130]
AQP7	Overexpressed	No data	Prostate cancer	[127,128]
AQP8	N/A	N/A	Cervical, gastric, pancreatic cancer	[130,136]
AQP9	Increased	No data	Brain, ovarian, prostate cancer	[120,121,127]

BC—breast cancer; N/A—not applicable.

**Table 3:** Aquaporins and breast cancer treatments.

Peroxiporin	Therapy and Expression	Interactions and/or Regulation	References
AQP1	Higher expression → sensitivity to anthracycline	β-catenin topoisomerase II Annexin A2 and Rab1b	[117,118,119]
AQP3	Higher expression → resistance to nucleotide analogs		[108,124,125,126]
AQP5	Upregulation AQP5 expression in doxorubicin-resistant variant	Ras regulation: NFkB, JNK, MAPK, Rac1	[126,131,132,133]

BC—breast cancer.

## References

[1] Agre P., Bonhivers M., Borgnia M.J. (1998). The Aquaporins, Blueprints for Cellular Plumbing Systems. J. Biol. Chem..

[2] Sui H., Han B.-G., Lee J.K., Walian P., Jap B.K. (2001). Structural Basis of Water-Specific Transport through the AQP1 Water Channel. Nature.

[3] Wu B., Beitz E. (2007). Aquaporins with Selectivity for Unconventional Permeants. Cell. Mol. Life Sci..

[4] de Maré S.W., Venskutonytė R., Eltschkner S., de Groot B.L., Lindkvist-Petersson K. (2020). Structural Basis for Glycerol Efflux and Selectivity of Human Aquaporin 7. Structure.

[5] Liu K., Kozono D., Kato Y., Agre P., Hazama A., Yasui M. (2005). Conversion of Aquaporin 6 from an Anion Channel to a Water-Selective Channel by a Single Amino Acid Substitution. Proc. Natl. Acad. Sci. USA.

[6] Bienert G.P., Møller A.L.B., Kristiansen K.A., Schulz A., Møller I.M., Schjoerring J.K., Jahn T.P. (2007). Specific Aquaporins Facilitate the Diffusion of Hydrogen Peroxide across Membranes. J. Biol. Chem..

[7] Prata C., Hrelia S., Fiorentini D. (2019). Peroxiporins in Cancer. Int. J. Mol. Sci..

[8] Soveral G., Nielsen S., Casini A. (2016). Aquaporins in Health and Disease.

[9] Bill R.M. (2024). Drugging Aquaporins. Biochim. Biophys. Acta (BBA) Biomembr..

[10] Obidiro O., Battogtokh G., Akala E.O. (2023). Triple Negative Breast Cancer Treatment Options and Limitations: Future Outlook. Pharmaceutics.

[11] Jusu S.M., Obayemi J.D., Salifu A.A., Nwazojie C.C., Uzonwanne V., Odusanya O.S., Soboyejo W.O. (2020). Drug-Encapsulated Blend of PLGA-PEG Microspheres: In Vitro and in Vivo Study of the Effects of Localized/Targeted Drug Delivery on the Treatment of Triple-Negative Breast Cancer. Sci. Rep..

[12] Neves Rebello Alves L., Dummer Meira D., Poppe Merigueti L., Correia Casotti M., do Prado Ventorim D., Ferreira Figueiredo Almeida J., Pereira de Sousa V., Cindra Sant’Ana M., Gonçalves Coutinho da Cruz R., Santos Louro L. (2023). Biomarkers in Breast Cancer: An Old Story with a New End. Genes.

[13] Menon S.S., Guruvayoorappan C., Sakthivel K.M., Rasmi R.R. (2019). Ki-67 Protein as a Tumour Proliferation Marker. Clin. Chim. Acta.

[14] Loizides S., Constantinidou A. (2023). Triple Negative Breast Cancer: Immunogenicity, Tumor Microenvironment, and Immunotherapy. Front. Genet..

[15] Neiger H.E., Siegler E.L., Shi Y. (2021). Breast Cancer Predisposition Genes and Synthetic Lethality. Int. J. Mol. Sci..

[16] Cardoso F., Kyriakides S., Ohno S., Penault-Llorca F., Poortmans P., Rubio I.T., Zackrisson S., Senkus E. (2019). Early Breast Cancer: ESMO Clinical Practice Guidelines for Diagnosis, Treatment and Follow-Up. Ann. Oncol..

[17] Wang D.-Y., Jiang Z., Ben-David Y., Woodgett J.R., Zacksenhaus E. (2019). Molecular Stratification within Triple-Negative Breast Cancer Subtypes. Sci. Rep..

[18] Yin L., Duan J.-J., Bian X.-W., Yu S. (2020). Triple-Negative Breast Cancer Molecular Subtyping and Treatment Progress. Breast Cancer Res..

[19] Almansour N.M. (2022). Triple-Negative Breast Cancer: A Brief Review About Epidemiology, Risk Factors, Signaling Pathways, Treatment and Role of Artificial Intelligence. Front. Mol. Biosci..

[20] Zheng Y., Li S., Tang H., Meng X., Zheng Q. (2023). Molecular Mechanisms of Immunotherapy Resistance in Triple-Negative Breast Cancer. Front. Immunol..

[21] Burstein M.D., Tsimelzon A., Poage G.M., Covington K.R., Contreras A., Fuqua S.A.W., Savage M.I., Osborne C.K., Hilsenbeck S.G., Chang J.C. (2015). Comprehensive Genomic Analysis Identifies Novel Subtypes and Targets of Triple-Negative Breast Cancer. Clin. Cancer Res..

[22] Lehmann B.D., Jovanović B., Chen X., Estrada M.V., Johnson K.N., Shyr Y., Moses H.L., Sanders M.E., Pietenpol J.A. (2016). Refinement of Triple-Negative Breast Cancer Molecular Subtypes: Implications for Neoadjuvant Chemotherapy Selection. PLoS ONE.

[23] Geyer C.E., Garber J.E., Gelber R.D., Yothers G., Taboada M., Ross L., Rastogi P., Cui K., Arahmani A., Aktan G. (2022). Overall Survival in the OlympiA Phase III Trial of Adjuvant Olaparib in Patients with Germline Pathogenic Variants in BRCA1/2 and High-Risk, Early Breast Cancer. Ann. Oncol..

[24] Deepak K.G.K., Vempati R., Nagaraju G.P., Dasari V.R., Nagini S., Rao D.N., Malla R.R. (2020). Tumor Microenvironment: Challenges and Opportunities in Targeting Metastasis of Triple Negative Breast Cancer. Pharmacol. Res..

[25] Mazzitelli C., Santini D., Corradini A.G., Zamagni C., Trerè D., Montanaro L., Taffurelli M. (2023). Liquid Biopsy in the Management of Breast Cancer Patients: Where Are We Now and Where Are We Going. Diagnostics.

[26] Perou C.M., Sørile T., Eisen M.B., Rijn M.V.D., Jeffrey S.S., Ress C.A., Pollack J.R., Ross D.T., Johnsen H., Akslen L.A. (2000). Molecular Portraits of Human Breast Tumours. Nature.

[27] Gradishar W.J., Moran M.S., Abraham J., Abramson V., Aft R., Agnese D., Allison K.H., Anderson B., Burstein H.J., Chew H. (2023). NCCN Guidelines^®^ Insights: Breast Cancer, Version 4.2023. J. Natl. Compr. Canc Netw..

[28] Early Breast Cancer Trialists’ Collaborative Group (EBCTCG) (2005). Effects of Chemotherapy and Hormonal Therapy for Early Breast Cancer on Recurrence and 15-Year Survival: An Overview of the Randomised Trials. Lancet.

[29] Juul N., Szallasi Z., Eklund A.C., Li Q., Burrell R.A., Gerlinger M., Valero V., Andreopoulou E., Esteva F.J., Symmans W.F. (2010). Assessment of an RNA Interference Screen-Derived Mitotic and Ceramide Pathway Metagene as a Predictor of Response to Neoadjuvant Paclitaxel for Primary Triple-Negative Breast Cancer: A Retrospective Analysis of Five Clinical Trials. Lancet Oncol..

[30] Bauer J.A., Chakravarthy A.B., Rosenbluth J.M., Mi D., Seeley E.H., De Matos Granja-Ingram N., Olivares M.G., Kelley M.C., Mayer I.A., Meszoely I.M. (2010). Identification of Markers of Taxane Sensitivity Using Proteomic and Genomic Analyses of Breast Tumors from Patients Receiving Neoadjuvant Paclitaxel and Radiation. Clin. Cancer Res..

[31] Loibl S., O’Shaughnessy J., Untch M., Sikov W.M., Rugo H.S., McKee M.D., Huober J., Golshan M., von Minckwitz G., Maag D. (2018). Addition of the PARP Inhibitor Veliparib plus Carboplatin or Carboplatin Alone to Standard Neoadjuvant Chemotherapy in Triple-Negative Breast Cancer (BrighTNess): A Randomised, Phase 3 Trial. Lancet Oncol..

[32] Sikov W.M., Berry D.A., Perou C.M., Singh B., Cirrincione C.T., Tolaney S.M., Kuzma C.S., Pluard T.J., Somlo G., Port E.R. (2015). Impact of the Addition of Carboplatin and/or Bevacizumab to Neoadjuvant Once-per-Week Paclitaxel Followed by Dose-Dense Doxorubicin and Cyclophosphamide on Pathologic Complete Response Rates in Stage II to III Triple-Negative Breast Cancer: CALGB 40603 (Alliance). J. Clin. Oncol..

[33] von Minckwitz G., Schneeweiss A., Loibl S., Salat C., Denkert C., Rezai M., Blohmer J.U., Jackisch C., Paepke S., Gerber B. (2014). Neoadjuvant Carboplatin in Patients with Triple-Negative and HER2-Positive Early Breast Cancer (GeparSixto; GBG 66): A Randomised Phase 2 Trial. Lancet Oncol..

[34] Tutt A., Tovey H., Cheang M.C.U., Kernaghan S., Kilburn L., Gazinska P., Owen J., Abraham J., Barrett S., Barrett-Lee P. (2018). Carboplatin in BRCA1/2-Mutated and Triple-Negative Breast Cancer BRCAness Subgroups: The TNT Trial. Nat. Med..

[35] Lehmann B.D., Bauer J.A., Chen X., Sanders M.E., Chakravarthy A.B., Shyr Y., Pietenpol J.A. (2011). Identification of Human Triple-Negative Breast Cancer Subtypes and Preclinical Models for Selection of Targeted Therapies. J. Clin. Investig..

[36] Geyer C.E., Sikov W.M., Huober J., Rugo H.S., Wolmark N., O’Shaughnessy J., Maag D., Untch M., Golshan M., Lorenzo J.P. (2022). Long-Term Efficacy and Safety of Addition of Carboplatin with or without Veliparib to Standard Neoadjuvant Chemotherapy in Triple-Negative Breast Cancer: 4-Year Follow-up Data from BrighTNess, a Randomized Phase III Trial. Ann. Oncol..

[37] Boughey J.C., Alvarado M.D., Lancaster R.B., Symmans W.F., Mukhtar R., Wong J.M., Ewing C.A., Potter D.A., Tuttle T.M., Hieken T.J. (2019). Erratum: Author Correction: Surgical Standards for Management of the Axilla in Breast Cancer Clinical Trials with Pathological Complete Response Endpoint. NPJ Breast Cancer.

[38] Piltin M.A., Hoskin T.L., Day C.N., Davis J., Boughey J.C. (2020). Oncologic Outcomes of Sentinel Lymph Node Surgery After Neoadjuvant Chemotherapy for Node-Positive Breast Cancer. Ann. Surg. Oncol..

[39] Tutt A.N.J., Garber J.E., Kaufman B., Viale G., Fumagalli D., Rastogi P., Gelber R.D., de Azambuja E., Fielding A., Balmaña J. (2021). Adjuvant Olaparib for Patients with BRCA1- or BRCA2-Mutated Breast Cancer. N. Engl. J. Med..

[40] Masuda N., Lee S.-J., Ohtani S., Im Y.-H., Lee E.-S., Yokota I., Kuroi K., Im S.-A., Park B.-W., Kim S.-B. (2017). Adjuvant Capecitabine for Breast Cancer after Preoperative Chemotherapy. N. Engl. J. Med..

[41] Yau C., Osdoit M., van der Noordaa M., Shad S., Wei J., de Croze D., Hamy A.-S., Laé M., Reyal F., Sonke G.S. (2022). Residual Cancer Burden after Neoadjuvant Chemotherapy and Long-Term Survival Outcomes in Breast Cancer: A Multicentre Pooled Analysis of 5161 Patients. Lancet Oncol..

[42] Symmans W.F., Wei C., Gould R., Yu X., Zhang Y., Liu M., Walls A., Bousamra A., Ramineni M., Sinn B. (2017). Long-Term Prognostic Risk After Neoadjuvant Chemotherapy Associated with Residual Cancer Burden and Breast Cancer Subtype. J. Clin. Oncol..

[43] Boughey J.C., Ballman K.V., McCall L.M., Mittendorf E.A., Symmans W.F., Julian T.B., Byrd D., Hunt K.K. (2017). Tumor Biology and Response to Chemotherapy Impact Breast Cancer-Specific Survival in Node-Positive Breast Cancer Patients Treated with Neoadjuvant Chemotherapy: Long-Term Follow-up from ACOSOG Z1071 (Alliance). Ann. Surg..

[44] Symmans W.F., Peintinger F., Hatzis C., Rajan R., Kuerer H., Valero V., Assad L., Poniecka A., Hennessy B., Green M. (2007). Measurement of Residual Breast Cancer Burden to Predict Survival After Neoadjuvant Chemotherapy. J. Clin. Oncol..

[45] Cortazar P., Zhang L., Untch M., Mehta K., Costantino J.P., Wolmark N., Bonnefoi H., Cameron D., Gianni L., Valagussa P. (2014). Pathological Complete Response and Long-Term Clinical Benefit in Breast Cancer: The CTNeoBC Pooled Analysis. Lancet.

[46] Luen S.J., Salgado R., Dieci M.V., Vingiani A., Curigliano G., Gould R.E., Castaneda C., D’Alfonso T., Sanchez J., Cheng E. (2019). Prognostic Implications of Residual Disease Tumor-Infiltrating Lymphocytes and Residual Cancer Burden in Triple-Negative Breast Cancer Patients after Neoadjuvant Chemotherapy. Ann. Oncol..

[47] Dieci M.V., Criscitiello C., Goubar A., Viale G., Conte P., Guarneri V., Ficarra G., Mathieu M.C., Delaloge S., Curigliano G. (2014). Prognostic Value of Tumor-Infiltrating Lymphocytes on Residual Disease after Primary Chemotherapy for Triple-Negative Breast Cancer: A Retrospective Multicenter Study. Ann. Oncol..

[48] Sun W.Y., Lee Y.K., Koo J.S. (2016). Expression of PD-L1 in Triple-Negative Breast Cancer Based on Different Immunohistochemical Antibodies. J. Transl. Med..

[49] Pardoll D.M. (2012). The Blockade of Immune Checkpoints in Cancer Immunotherapy. Nat. Rev. Cancer.

[50] Chen D.S., Mellman I. (2013). Oncology Meets Immunology: The Cancer-Immunity Cycle. Immunity.

[51] Nanda R., Liu M.C., Yau C., Shatsky R., Pusztai L., Wallace A., Chien A.J., Forero-Torres A., Ellis E., Han H. (2020). Effect of Pembrolizumab Plus Neoadjuvant Chemotherapy on Pathologic Complete Response in Women With Early-Stage Breast Cancer: An Analysis of the Ongoing Phase 2 Adaptively Randomized I-SPY2 Trial. JAMA Oncol..

[52] Schmid P., Cortes J., Dent R., Pusztai L., McArthur H., Kümmel S., Bergh J., Denkert C., Park Y.H., Hui R. (2022). Event-Free Survival with Pembrolizumab in Early Triple-Negative Breast Cancer. N. Engl. J. Med..

[53] Cortes J., Cescon D.W., Rugo H.S., Nowecki Z., Im S.-A., Yusof M.M., Gallardo C., Lipatov O., Barrios C.H., Holgado E. (2020). Pembrolizumab plus Chemotherapy versus Placebo plus Chemotherapy for Previously Untreated Locally Recurrent Inoperable or Metastatic Triple-Negative Breast Cancer (KEYNOTE-355): A Randomised, Placebo-Controlled, Double-Blind, Phase 3 Clinical Trial. Lancet.

[54] Mittendorf E.A., Zhang H., Barrios C.H., Saji S., Jung K.H., Hegg R., Koehler A., Sohn J., Iwata H., Telli M.L. (2020). Neoadjuvant Atezolizumab in Combination with Sequential Nab-Paclitaxel and Anthracycline-Based Chemotherapy versus Placebo and Chemotherapy in Patients with Early-Stage Triple-Negative Breast Cancer (IMpassion031): A Randomised, Double-Blind, Phase 3 Trial. Lancet.

[55] Stringer-Reasor E., Shatsky R.A., Chien J., Wallace A., Boughey J.C., Albain K.S., Han H.S., Nanda R., Isaacs C., Kalinsky K. (2023). Abstract PD11-01: PD11-01 Evaluation of the PD-1 Inhibitor Cemiplimab in Early-Stage, High-Risk HER2-Negative Breast Cancer: Results from the Neoadjuvant I-SPY 2 TRIAL. Cancer Res..

[56] Loibl S., Schneeweiss A., Huober J., Braun M., Rey J., Blohmer J.-U., Furlanetto J., Zahm D.-M., Hanusch C., Thomalla J. (2022). Neoadjuvant Durvalumab Improves Survival in Early Triple-Negative Breast Cancer Independent of Pathological Complete Response. Ann. Oncol..

[57] Couch F.J., Hart S.N., Sharma P., Toland A.E., Wang X., Miron P., Olson J.E., Godwin A.K., Pankratz V.S., Olswold C. (2015). Inherited Mutations in 17 Breast Cancer Susceptibility Genes among a Large Triple-Negative Breast Cancer Cohort Unselected for Family History of Breast Cancer. J. Clin. Oncol..

[58] Singh D.D., Parveen A., Yadav D.K. (2021). Role of PARP in TNBC: Mechanism of Inhibition, Clinical Applications, and Resistance. Biomedicines.

[59] Krop I., Juric D., Shimizu T., Tolcher A., Spira A., Mukohara T., Lisberg A.E., Kogawa T., Papadopoulos K.P., Hamilton E. (2022). Abstract GS1-05: Datopotamab Deruxtecan in Advanced/Metastatic HER2- Breast Cancer: Results from the Phase 1 TROPION-PanTumor01 Study. Cancer Res..

[60] Krop I.E., Masuda N., Mukohara T., Takahashi S., Nakayama T., Inoue K., Iwata H., Toyama T., Yamamoto Y., Hansra D.M. (2022). Results from the Phase 1/2 Study of Patritumab Deruxtecan, a HER3-Directed Antibody-Drug Conjugate (ADC), in Patients with HER3-Expressing Metastatic Breast Cancer (MBC). J. Clin. Oncol..

[61] Commissioner of the U.S. Food and Drug Administration. https://www.fda.gov/.

[62] Homepage|European Medicines Agency. https://www.ema.europa.eu/en/homepage.

[63] Schmid P., Abraham J., Chan S., Wheatley D., Brunt A.M., Nemsadze G., Baird R.D., Park Y.H., Hall P.S., Perren T. (2020). Capivasertib Plus Paclitaxel Versus Placebo Plus Paclitaxel as First-Line Therapy for Metastatic Triple-Negative Breast Cancer: The PAKT Trial. J. Clin. Oncol..

[64] Leon-Ferre R.A., Goetz M.P. (2023). Advances in Systemic Therapies for Triple Negative Breast Cancer. BMJ.

[65] Jézéquel P., Kerdraon O., Hondermarck H., Guérin-Charbonnel C., Lasla H., Gouraud W., Canon J.-L., Gombos A., Dalenc F., Delaloge S. (2019). Identification of Three Subtypes of Triple-Negative Breast Cancer with Potential Therapeutic Implications. Breast Cancer Res..

[66] Thompson K.J., Leon-Ferre R.A., Sinnwell J.P., Zahrieh D.M., Suman V.J., Metzger F.O., Asad S., Stover D.G., Carey L., Sikov W.M. (2022). Luminal Androgen Receptor Breast Cancer Subtype and Investigation of the Microenvironment and Neoadjuvant Chemotherapy Response. NAR Cancer.

[67] Vinayak S., Tolaney S.M., Schwartzberg L., Mita M., McCann G., Tan A.R., Wahner-Hendrickson A.E., Forero A., Anders C., Wulf G.M. (2019). Open-Label Clinical Trial of Niraparib Combined with Pembrolizumab for Treatment of Advanced or Metastatic Triple-Negative Breast Cancer. JAMA Oncol..

[68] Azad A.K., Raihan T., Ahmed J., Hakim A., Emon T.H., Chowdhury P.A. (2021). Human Aquaporins: Functional Diversity and Potential Roles in Infectious and Non-Infectious Diseases. Front. Genet..

[69] Čipak Gašparović A., Milković L., Rodrigues C., Mlinarić M., Soveral G. (2021). Peroxiporins Are Induced upon Oxidative Stress Insult and Are Associated with Oxidative Stress Resistance in Colon Cancer Cell Lines. Antioxidants.

[70] Kourghi M., Pei J.V., Ieso M.L.D., Nourmohammadi S., Chow P.H., Yool A.J. (2018). Fundamental Structural and Functional Properties of Aquaporin Ion Channels Found across the Kingdoms of Life. Clin. Exp. Pharmacol. Physiol..

[71] Direito I., Madeira A., Brito M.A., Soveral G. (2016). Aquaporin-5: From Structure to Function and Dysfunction in Cancer. Cell Mol. Life Sci..

[72] Wang Y., Tajkhorshid E. (2007). Molecular Mechanisms of Conduction and Selectivity in Aquaporin Water Channels123. J. Nutr..

[73] de Groot B.L., Grubmüller H. (2005). The Dynamics and Energetics of Water Permeation and Proton Exclusion in Aquaporins. Curr. Opin. Struct. Biol..

[74] Jung J.S., Preston G.M., Smith B.L., Guggino W.B., Agre P. (1994). Molecular Structure of the Water Channel through Aquaporin CHIP. The Hourglass Model. J. Biol. Chem..

[75] Gravelle S., Joly L., Detcheverry F., Ybert C., Cottin-Bizonne C., Bocquet L. (2013). Optimizing Water Permeability through the Hourglass Shape of Aquaporins. Proc. Natl. Acad. Sci. USA.

[76] Hub J.S., de Groot B.L. (2008). Mechanism of Selectivity in Aquaporins and Aquaglyceroporins. Proc. Natl. Acad. Sci. USA.

[77] Beitz E., Wu B., Holm L.M., Schultz J.E., Zeuthen T. (2006). Point Mutations in the Aromatic/Arginine Region in Aquaporin 1 Allow Passage of Urea, Glycerol, Ammonia, and Protons. Proc. Natl. Acad. Sci. USA.

[78] Hub J.S., de Groot B.L. (2006). Does CO_2_ Permeate through Aquaporin-1?. Biophys. J..

[79] Bollag W.B., Aitkens L., White J., Hyndman K.A. (2020). Aquaporin-3 in the Epidermis: More than Skin Deep. Am. J. Physiol. Cell Physiol..

[80] Fotiadis D., Suda K., Tittmann P., Jenö P., Philippsen A., Müller D.J., Gross H., Engel A. (2002). Identification and Structure of a Putative Ca^2+^-Binding Domain at the C Terminus of AQP1. J. Mol. Biol..

[81] Smith B.L., Agre P. (1991). Erythrocyte Mr 28,000 Transmembrane Protein Exists as a Multisubunit Oligomer Similar to Channel Proteins. J. Biol. Chem..

[82] Kitchen P., Salman M.M., Pickel S.U., Jennings J., Törnroth-Horsefield S., Conner M.T., Bill R.M., Conner A.C. (2019). Water Channel Pore Size Determines Exclusion Properties but Not Solute Selectivity. Sci. Rep..

[83] Zeuthen T., Klaerke D.A. (1999). Transport of Water and Glycerol in Aquaporin 3 Is Gated by H+. J. Biol. Chem..

[84] Zelenina M., Bondar A.A., Zelenin S., Aperia A. (2003). Nickel and Extracellular Acidification Inhibit the Water Permeability of Human Aquaporin-3 in Lung Epithelial Cells. J. Biol. Chem..

[85] Almeida A.D., Martins A.P., Mósca A.F., Wijma H.J., Prista C., Soveral G., Casini A. (2016). Exploring the Gating Mechanisms of Aquaporin-3: New Clues for the Design of Inhibitors?. Mol. BioSyst..

[86] Zelenina M., Tritto S., Bondar A.A., Zelenin S., Aperia A. (2004). Copper Inhibits the Water and Glycerol Permeability of Aquaporin-3. J. Biol. Chem..

[87] Horsefield R., Nordén K., Fellert M., Backmark A., Törnroth-Horsefield S., Terwisscha van Scheltinga A.C., Kvassman J., Kjellbom P., Johanson U., Neutze R. (2008). High-Resolution x-Ray Structure of Human Aquaporin 5. Proc. Natl. Acad. Sci. USA.

[88] Raina S., Preston G.M., Guggino W.B., Agre P. (1995). Molecular Cloning and Characterization of an Aquaporin cDNA from Salivary, Lacrimal, and Respiratory Tissues. J. Biol. Chem..

[89] Woo J., Chae Y.K., Jang S.J., Kim M.S., Baek J.H., Park J.C., Trink B., Ratovitski E., Lee T., Park B. (2008). Membrane Trafficking of AQP5 and cAMP Dependent Phosphorylation in Bronchial Epithelium. Biochem. Biophys. Res. Commun..

[90] Kosugi-Tanaka C., Li X., Yao C., Akamatsu T., Kanamori N., Hosoi K. (2006). Protein Kinase A-Regulated Membrane Trafficking of a Green Fluorescent Protein-Aquaporin 5 Chimera in MDCK Cells. Biochim. Biophys. Acta.

[91] Woo J., Lee J., Kim M.S., Jang S.J., Sidransky D., Moon C. (2008). The Effect of Aquaporin 5 Overexpression on the Ras Signaling Pathway. Biochem. Biophys. Res. Commun..

[92] Ozu M., Galizia L., Acuña C., Amodeo G. (2018). Aquaporins: More Than Functional Monomers in a Tetrameric Arrangement. Cells.

[93] Charlestin V., Fulkerson D., Arias Matus C.E., Walker Z.T., Carthy K., Littlepage L.E. (2022). Aquaporins: New Players in Breast Cancer Progression and Treatment Response. Front. Oncol..

[94] Yu J., Yool A.J., Schulten K., Tajkhorshid E. (2006). Mechanism of Gating and Ion Conductivity of a Possible Tetrameric Pore in Aquaporin-1. Structure.

[95] Yool A.J., Weinstein A.M. (2002). New Roles for Old Holes: Ion Channel Function in Aquaporin-1. News Physiol. Sci..

[96] Wang Y., Tajkhorshid E. (2010). Nitric Oxide Conduction by the Brain Aquaporin AQP4. Proteins.

[97] Alishahi M., Kamali R. (2019). A Novel Molecular Dynamics Study of CO_2_ Permeation through Aquaporin-5. Eur. Phys. J. E.

[98] Edamana S., Login F.H., Yamada S., Kwon T.-H., Nejsum L.N. (2021). Aquaporin Water Channels as Regulators of Cell-Cell Adhesion Proteins. Am. J. Physiol. Cell Physiol..

[99] Ji Y., Liao X., Jiang Y., Wei W., Yang H. (2021). Aquaporin 1 Knockdown Inhibits Triple-Negative Breast Cancer Cell Proliferation and Invasion in Vitro and in Vivo. Oncol. Lett..

[100] Login F.H., Palmfeldt J., Cheah J.S., Yamada S., Nejsum L.N. (2021). Aquaporin-5 Regulation of Cell-Cell Adhesion Proteins: An Elusive “Tail” Story. Am. J. Physiology. Cell Physiol..

[101] Mobasheri A., Wray S., Marples D. (2005). Distribution of AQP2 and AQP3 Water Channels in Human Tissue Microarrays. J. Mol. Hist..

[102] Mobasheri A., Barrett-Jolley R. (2014). Aquaporin Water Channels in the Mammary Gland: From Physiology to Pathophysiology and Neoplasia. J. Mammary Gland. Biol. Neoplasia.

[103] Bystrup M., Login F.H., Edamana S., Borgquist S., Tramm T., Kwon T.H., Nejsum L.N. (2022). Aquaporin-5 in Breast Cancer. Apmis.

[104] Otterbach F., Callies R., Adamzik M., Kimmig R., Siffert W., Schmid K.W., Bankfalvi A. (2010). Aquaporin 1 (AQP1) Expression Is a Novel Characteristic Feature of a Particularly Aggressive Subgroup of Basal-like Breast Carcinomas. Breast Cancer Res. Treat..

[105] Kaneko K., Yagui K., Tanaka A., Yoshihara K., Ishikawa K., Takahashi K., Bujo H., Sakurai K., Saito Y. (2008). Aquaporin 1 Is Required for Hypoxia-Inducible Angiogenesis in Human Retinal Vascular Endothelial Cells. Microvasc. Res..

[106] Zhang B., Liu F., Ma Y., Gu F. (2013). Cytoplasmic expression of aquaporin-1 in breast cancer cells and its relationship with clinicopathological characteristics and prognosis. Zhonghua Zhong Liu Za Zhi.

[107] Mobasheri A., Airley R., Hewitt S.M., Marples D. (2005). Heterogeneous Expression of the Aquaporin 1 (AQP1) Water Channel in Tumors of the Prostate, Breast, Ovary, Colon and Lung: A Study Using High Density Multiple Human Tumor Tissue Microarrays. Int. J. Oncol..

[108] Huang Y.-T., Zhou J., Shi S., Xu H.-Y., Qu F., Zhang D., Chen Y.-D., Yang J., Huang H.-F., Sheng J.-Z. (2015). Identification of Estrogen Response Element in Aquaporin-3 Gene That Mediates Estrogen-Induced Cell Migration and Invasion in Estrogen Receptor-Positive Breast Cancer. Sci. Rep..

[109] Jung H.J., Park J.Y., Jeon H.S., Kwon T.H. (2011). Aquaporin-5: A Marker Protein for Proliferation and Migration of Human Breast Cancer Cells. PLoS ONE.

[110] Marlar S., Jensen H.H., Login F.H., Nejsum L.N. (2017). Aquaporin-3 in Cancer. Int. J. Mol. Sci..

[111] Traberg-Nyborg L., Login F.H., Edamana S., Tramm T., Borgquist S., Nejsum L.N. (2022). Aquaporin-1 in Breast Cancer. APMIS.

[112] Conner M.T., Conner A.C., Bland C.E., Taylor L.H.J., Brown J.E.P., Parri H.R., Bill R.M. (2012). Rapid Aquaporin Translocation Regulates Cellular Water Flow. J. Biol. Chem..

[113] Login F.H., Jensen H.H., Pedersen G.A., Koffman J.S., Kwon T.-H., Parsons M., Nejsum L.N. (2019). Aquaporins Differentially Regulate Cell-Cell Adhesion in MDCK Cells. FASEB J..

[114] Conner M.T., Conner A.C., Brown J.E.P., Bill R.M. (2010). Membrane Trafficking of Aquaporin 1 Is Mediated by Protein Kinase C via Microtubules and Regulated by Tonicity. Biochemistry.

[115] He S., Li Q., Huang Q., Cheng J. (2022). Targeting Protein Kinase C for Cancer Therapy. Cancers.

[116] Wei M., Yu H., Cai C., Gao R., Liu X., Zhu H. (2020). MiR-3194-3p Inhibits Breast Cancer Progression by Targeting Aquaporin1. Front. Oncol..

[117] Chong W., Zhang H., Guo Z., Yang L., Shao Y., Liu X., Zhao Y., Wang Z., Zhang M., Guo C. (2021). Aquaporin 1 Promotes Sensitivity of Anthracycline Chemotherapy in Breast Cancer by Inhibiting β-Catenin Degradation to Enhance TopoIIα Activity. Cell Death Differ..

[118] Guo Z., Zhang H., Liu X., Zhao Y., Chen Y., Jin J., Guo C., Zhang M., Gu F., Ma Y. (2023). Water Channel Protein AQP1 in Cytoplasm Is a Critical Factor in Breast Cancer Local Invasion. J. Exp. Clin. Cancer Res. CR.

[119] Qin F., Zhang H., Shao Y., Liu X., Yang L., Huang Y., Fu L., Gu F., Ma Y. (2016). Expression of Aquaporin1, a Water Channel Protein, in Cytoplasm Is Negatively Correlated with Prognosis of Breast Cancer Patients. Oncotarget.

[120] Kasa P., Farran B., Prasad G.L.V., Nagaraju G.P. (2019). Aquaporins in Female Specific Cancers. Gene.

[121] Moon C.S., Moon D., Kang S.K. (2022). Aquaporins in Cancer Biology. Front. Oncol..

[122] Imaizumi H., Ishibashi K., Takenoshita S., Ishida H. (2018). Aquaporin 1 Expression Is Associated with Response to Adjuvant Chemotherapy in Stage II and III Colorectal Cancer. Oncol. Lett..

[123] Bruun-Sørensen A.S., Edamana S., Login F.H., Borgquist S., Nejsum L.N. (2021). Aquaporins in Pancreatic Ductal Adenocarcinoma. APMIS.

[124] Trigueros-Motos L., Pérez-Torras S., Casado F.J., Molina-Arcas M., Pastor-Anglada M. (2012). Aquaporin 3 (AQP3) Participates in the Cytotoxic Response to Nucleoside-Derived Drugs. BMC Cancer.

[125] Arif M., Kitchen P., Conner M.T., Hill E.J., Nagel D., Bill R.M., Dunmore S.J., Armesilla A.L., Gross S., Carmichael A.R. (2018). Downregulation of Aquaporin 3 Inhibits Cellular Proliferation, Migration and Invasion in the MDA-MB-231 Breast Cancer Cell Line. Oncol. Lett..

[126] Zhu Z., Jiao L., Li T., Wang H., Wei W., Qian H. (2018). Expression of AQP3 and AQP5 as a Prognostic Marker in Triple-Negative Breast Cancer. Oncol. Lett..

[127] Kirkegaard T., Riishede A., Tramm T., Nejsum L.N. (2023). Aquaglyceroporins in Human Breast Cancer. Cells.

[128] Kushwaha P.P., Verma S., Gupta S. (2023). Aquaporins as Prognostic Biomarker in Prostate Cancer. Cancers.

[129] Saadoun S., Papadopoulos M.C., Davies D.C., Krishna S., Bell B.A. (2002). Aquaporin-4 Expression Is Increased in Oedematous Human Brain Tumours. J. Neurol. Neurosurg. Psychiatry.

[130] Thapa S., Chetry M., Huang K., Peng Y., Wang J., Wang J., Zhou Y., Shen Y., Xue Y., Ji K. (2018). Significance of Aquaporins’ Expression in the Prognosis of Gastric Cancer. Biosci. Rep..

[131] Li X., Pei B., Wang H., Tang C., Zhu W., Jin F. (2018). Effect of AQP-5 Silencing by siRNA Interference on Chemosensitivity of Breast Cancer Cells. OncoTargets Ther..

[132] Edamana S., Pedersen S.F., Nejsum L.N. (2023). Aquaporin Water Channels Affect the Response of Conventional Anticancer Therapies of 3D Grown Breast Cancer Cells. Biochem. Biophys. Res. Commun..

[133] Bosco E.E., Mulloy J.C., Zheng Y. (2009). Rac1 GTPase: A “Rac” of All Trades. Cell Mol. Life Sci..

[134] Zhang T., Zhao C., Chen D., Zhou Z. (2012). Overexpression of AQP5 in Cervical Cancer: Correlation with Clinicopathological Features and Prognosis. Med. Oncol..

[135] Pust A., Kylies D., Hube-Magg C., Kluth M., Minner S., Koop C., Grob T., Graefen M., Salomon G., Tsourlakis M.C. (2016). Aquaporin 5 Expression Is Frequent in Prostate Cancer and Shows a Dichotomous Correlation with Tumor Phenotype and PSA Recurrence. Hum. Pathol..

[136] Li W., Song Y., Pan C., Yu J., Zhang J., Zhu X. (2021). Aquaporin-8 Is a Novel Marker for Progression of Human Cervical Cancer Cells. Cancer Biomark..

[137] Zou L.-B., Shi S., Zhang R.-J., Wang T.-T., Tan Y.-J., Zhang D., Fei X.-Y., Ding G.-L., Gao Q., Chen C. (2013). Aquaporin-1 Plays a Crucial Role in Estrogen-Induced Tubulogenesis of Vascular Endothelial Cells. J. Clin. Endocrinol. Metab..

[138] Dai C., Charlestin V., Wang M., Walker Z.T., Miranda-Vergara M.C., Facchine B.A., Wu J., Kaliney W.J., Dovichi N.J., Li J. (2020). Aquaporin-7 Regulates the Response to Cellular Stress in Breast Cancer. Cancer Res..

[139] Jang S.J., Moon C. (2023). Aquaporin 5 (AQP5) Expression in Breast Cancer and Its Clinicopathological Characteristics. PLoS ONE.

[140] Shi Z., Zhang T., Luo L., Zhao H., Cheng J., Xiang J., Zhao C. (2012). Aquaporins in Human Breast Cancer: Identification and Involvement in Carcinogenesis of Breast Cancer. J. Surg. Oncol..

[141] Lee S.J., Chae Y.S., Kim J.G., Kim W.W., Jung J.H., Park H.Y., Jeong J.Y., Park J.Y., Jung H.J., Kwon T.H. (2014). AQP5 Expression Predicts Survival in Patients with Early Breast Cancer. Ann. Surg. Oncol..

[142] Li Q., Yang T., Li D., Ding F., Bai G., Wang W., Sun H. (2018). Knockdown of Aquaporin-5 Sensitizes Colorectal Cancer Cells to 5-Fluorouracil via Inhibition of the Wnt–β-Catenin Signaling Pathway. Biochem. Cell Biol..

